# Replacing Computed Tomography with “Rapid” Magnetic Resonance Imaging for Ventricular Shunt Imaging

**DOI:** 10.1097/pq9.0000000000000441

**Published:** 2021-07-28

**Authors:** Jennifer R. Marin, Elizabeth C. Tyler-Kabara, Casey Anderson, Gabriella Butler, Shaquille Charles, Andre Furtado, Johanna R. Rosen

**Affiliations:** From the * Division of Pediatric Emergency Medicine, UPMC Children’s Hospital of Pittsburgh, Pittsburgh, Pa.; † Division of Pediatric Radiology, UPMC Children’s Hospital of Pittsburgh, Pittsburgh, Pa.; ‡ Division of Pediatric Neurosurgery, Dell Children’s Hospital, Austin, Tex.; § Division of Health Informatics,UPMC Children’s Hospital of Pittsburgh, Pittsburgh, Pa.; ¶ University of Pittsburgh School of Medicine, Pittsburgh,Pa.

## Abstract

**Introduction:**

Children with ventricular shunts undergo frequent neuroimaging, and therefore, radiation exposures, to evaluate shunt malfunctions. The objective of this study was to safely reduce radiation exposure in this population by reducing computed tomography (CT) and increasing “rapid” magnetic resonance imaging (rMRI-shunt) among patients warranting neuroimaging for possible shunt malfunction.

**Methods:**

This was a single-center quality improvement study in a tertiary care pediatric emergency department (ED). We implemented a multidisciplinary guideline for ED shunt evaluation, which promoted the use of rMRI-shunt over CT. We included patients younger than 18 years undergoing an ED shunt evaluation during 11 months of the preintervention and 25 months of the intervention study periods. The primary outcome was the CT rate, and we evaluated the relevant process and balancing measures.

**Results:**

There were 266 encounters preintervention and 488 during the intervention periods with similar neuroimaging rates (80.7% versus 81.5%, *P* = 0.8.) CT decreased from 90.1% to 34.8% (difference −55.3%, 95% confidence interval [CI]: −71.1, −25.8), and rMRI-shunt increased from 9.9% to 65.2% (difference 55.3%, 95% CI: 25.8, 71.1) during the preintervention and intervention periods, respectively. There were increases in the mean time to neuroimaging (53.1 min; [95% CI: 41.6, 64.6]) and ED length of stay (LOS) (52.3 min; [95% CI: 36.8, 67.6]), without changes in total neuroimaging, 72-hour revisits, or follow-up neuroimaging.

**Conclusions:**

Multidisciplinary implementation of a standardized guideline reduced CT and increased rMRI-shunt use in a pediatric ED setting. Clinicians should balance the reduction in radiation exposure with ED rMRI-shunt for patients with ventricular shunts against the increased time of obtaining imaging and LOS.

## Abstract 2

Supplemental Digital Content is available in the text.

## INTRODUCTION

Children with ventricular shunts require neuroimaging to evaluate for ventricular shunt malfunction. Historically, the primary neuroimaging modality to evaluate for shunt malfunction was computed tomography (CT).^1^ Although accurate, fast, and usually readily available in the emergency department (ED) setting, CT exposes pediatric patients to radiation, which may increase the lifetime risk of malignancy.^2,3^ Given the high risk of and nonspecific symptomatology associated with shunt failure,^4–6^ clinicians have a low threshold to perform neuroimaging. Prior work has demonstrated a significant number of CT exposures to pediatric patients with shunts over the lifetime of the shunt,^7–9^ which may result in a large cumulative radiation exposure.^10^

In the past decade, “fast” or “rapid” magnetic resonance imaging (rMRI-shunt) has emerged as an alternative to CT for pediatric neuroimaging.^11–14^ The most widely studied protocol has been that for ventricular shunt evaluation, with data demonstrating comparable accuracy between CT and rMRI-shunt for evaluating ventriculomegaly.^15,16^ Despite the diagnostic performance and radiation-sparing nature, rMRI-shunt examinations are not as readily available to clinicians, take more time than CT examinations, and young children may not tolerate even these “rapid” studies.

In our ED, the baseline rate of CT imaging among children with ventricular shunts undergoing neuroimaging was over 80%. To safely reduce radiation exposure to these patients, our objective for this study was to replace CT with rMRI-shunt as the primary neuroimaging study for the assessment of patients with suspected shunt malfunction. Specifically, the primary aim was to reduce head CTs by 40% within 1 year.

## PATIENTS AND METHODS

### Setting

We conducted a single-center quality improvement (QI) project in a pediatric ED within a tertiary care children’s hospital using SQUIRE 2.0 guidelines for publication of QI projects. The study ED has an annual volume of approximately 85,000 patients, is part of a 42-hospital health system and is the only pediatric ED in the region. The study ED, located on the hospital’s first floor, has a dedicated CT scanner available 24 hours/d, 7 days/wk. The hospital’s 4 MRI scanners are located on the second floor of the hospital. Technicians are present in the hospital and examinations are available 24 hours/d, 7 days/wk; however, there are fewer MRI technicians in the evenings and on weekends. Technicians perform scheduled outpatient examinations during the day, inpatient examinations throughout the day and evening, and emergent examinations from acute care areas (eg, ED and ICUs) around the clock. Although a 7-minute rMRI-shunt protocol for shunt evaluation (**Table, Supplemental Digital Content 1,** which displays rapid MRI ventricular shunt imaging protocol, http://links.lww.com/PQ9/A277) was available before implementing the QI intervention, clinicians rarely utilized it.

### Patient Cohort

This study involved patients younger than 18 years of age evaluated in the ED for possible shunt malfunction from April 1, 2017, to March 31, 2020. We used the following parameters to identify patients with possible shunt malfunction: (1) chief complaint of *shunt malfunction* (assigned upon arrival to the ED by a triage nurse from a standardized list of 110 complaints); (2) International Classification of Diseases, 10th revision, Clinical Modification (ICD10-CM) principal discharge diagnosis code for shunt complication^17^; and/or (3) ventricular shunt radiograph series evaluation performed during the ED encounter (based on a “completed” order in the electronic medical record.) It was standard practice at the study ED to obtain ventricular shunt radiographs on all patients evaluated for shunt malfunctions. We excluded patients transferred into the ED from outside institutions, because imaging is often performed before transfer. We extracted data electronically through an institutional software program.

### Developing the Intervention

**Fig. 1.**
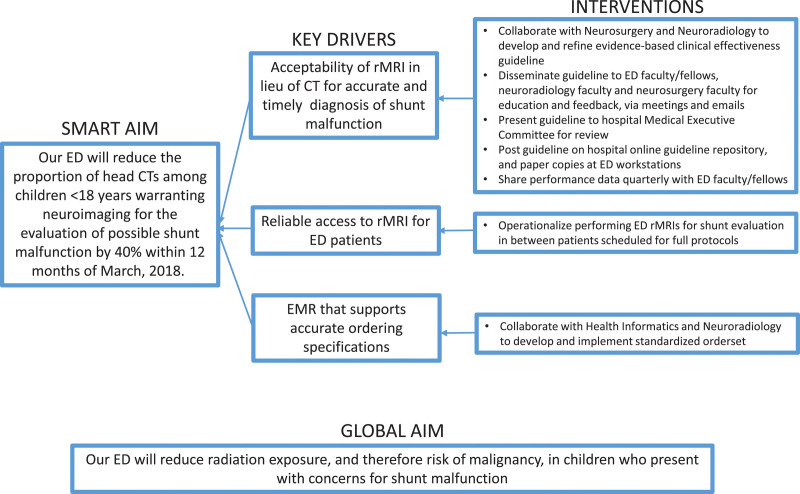
Key driver diagram for reducing head CT and increasing rMRI examinations for pediatric ED patients with suspected ventricular shunt malfunction. EMR, electronic medical record.

In July 2017, we assembled a multidisciplinary QI team to design, implement, and monitor the effect of a standardized evidence-based^15,16^ clinical effectiveness guideline for imaging ED patients with possible ventricular shunt malfunction. A pediatric emergency physician, pediatric neurosurgeon, pediatric neuroradiologist, and MRI technician comprised the group. Initially, the group identified key drivers (Fig. 1) and barriers to MRI use for ventricular shunt evaluation. Specific potential barriers identified were concerns regarding the time to obtain rMRI-shunt, adequate comparison of MRI to CT in patients who did not have any prior or recent MRIs, and the possible need for reprogramming a shunt at night when a neurosurgery resident was not in the hospital. The group reached a consensus that: (1) the division of pediatric neurosurgery would determine any contraindications to rMRI-shunt or scenarios when a CT would be preferable and (2) the division of pediatric neuroradiology would determine the potential frequency of rMRI-shunt orders for shunt evaluations and collaborate with MRI schedulers and technicians to ensure timely evaluations for ED patients. The neurosurgery group determined that only patients’ clinical status and potential delay in imaging would necessitate a CT instead of rMRI-shunt. Also, they advised that in rare cases when a patient with a programmable shunt underwent rMRI-shunt, required reprogramming, and the treating team did not consult a neurosurgery resident for in-person evaluation, the patient could be safely discharged and return to the clinic the next morning for reprogramming. The neuroradiology group determined there were approximately 185 ED neuroimaging studies performed in this population in the last year, and therefore, on average, one every other day; and, MRI technicians would make efforts to perform the rMRI-shunt examinations between already scheduled full brain MRI examinations to prioritize and expedite these ED evaluations.

The QI team drafted the clinical effectiveness guideline and revised it through an iterative process within the QI group. In March 2018, we presented the guideline to and solicited comments from the pediatric emergency medicine faculty and fellows, pediatric neurosurgery faculty, pediatric neuroradiologists, and the pediatric hospitalist group (**Figure, Supplemental Digital Content 2,** which describes clinical effectiveness guideline for ED neuroimaging in patients with possible ventricular shunt malfunction, http://links.lww.com/PQ9/A278). Additionally, we provided the guideline electronically to all relevant stakeholders, including pediatric residents and ED nursing leadership. This guideline presentation and feedback solicitation marked the beginning of our intervention study period because this outreach also functioned as an educational intervention about the benefits of MRI over CT and how we could begin replacing CT with MRI for shunt evaluations. In April 2018, the guideline was approved through the hospital’s Medical Executive Committee and published on the hospital’s online portal. Emails were sent regularly to ED providers and other relevant stakeholders (eg, neurosurgery and radiology faculty for dissemination to trainees) to improve engagement and compliance with the pathway. The principal investigator also shared updated data of neuroimaging patterns with ED faculty and fellows through quarterly emails. In July 2019, in collaboration with the information technology team members, we developed a specific ED MRI order set for the electronic health record (Cerner Corporation, Kansas City, Mo.) to facilitate ordering of rMRIs, as the hospital had multiple rMRI protocols for various indications. Before using this order set, physicians were relied upon to free-text “rapid shunt protocol” in the brain MRI noncontrast order to specify that protocol.

### Measures

As it is not feasible to measure malignancy incidence from CT radiation exposures, we chose to use a process measure (CT rate) as a proxy outcome measure. Additionally, we measured the following relevant process and balancing measures:

1. Time to neuroimaging, defined as ED room time to imaging completion;
2. ED LOS, defined as room time to final disposition (ie, discharge or admitted assignment made in the electronic health record);
3. Time to operative intervention, defined as room time to the surgical incision for patients who underwent surgery for shunt revision or replacement within 12 hours of ED arrival;
4. Total neuroimaging, defined as the number of CT and rMRI-shunt studies in the ED for discharged patients and the number of studies in the ED and within 24 hours for admitted patients;
5. 72-hour revisit, defined as a second ED visit for a shunt-related complaint/evaluation (as previously defined for patients in the cohort) within 72 hours of an index visit which resulted in discharge; and
6. Follow-up neuroimaging, defined as having any neuroimaging (noncontrast head CT and/or brain MRI [rapid or full brain]) within seven days of discharge from the ED (including inpatient and outpatient imaging at the study hospital).

If an ED encounter included both a CT and MRI, we counted the neuroimaging study that occurred first for all measures except total neuroimaging (both were counted). We evaluated the following patient demographics: age, sex, race/ethnicity, disposition from the ED, triage acuity at ED presentation as defined by the emergency severity index,^18^ time of day (8 am–5 pm versus 5 pm–8 am), and weekday versus weekend ED presentation, and primary payer. A patient or family member self-assigned race/ethnicity at the time of registration in the ED to 1 of 25 specified groups and we further categorized these 5 groups for ease of presentation (ie, White, Black, Hispanic, other, unknown).

### Data Analysis

We summarized patient characteristics during both the preintervention and intervention study periods using descriptive statistics and calculated CT and rMRI-shunt imaging proportions during both periods. We used the *t* test to compare continuous variables and the chi-squared test to compare categorical variables. We used a time-series design to assess the impact of the intervention. We assessed data 11 months before the intervention to establish a preguideline baseline and 25 months following pathway implementation to assess the guideline’s immediate and longer-term impact. We accounted for the data’s hierarchical nature (repeated visits by the same patient) using multilevel-longitudinal logistic regression modeling for the primary outcome.^19^ Statistical significance was set at *P* < 0.05. We used statistical process control (SPC) methods to monitor CT rate changes over time, with Shewhart rules for control limits (3 SDs) and significant deviations from the centerline.^20^ We used SigmaZone SPC-XL 2010 integrated into Microsoft Excel (SigmaZone, Windermere, Fla.) to create the statistical process charts. A statistical expert (S.C.) used Stata version 16.0 (StataCorp LLC, College Station, Tex.) for all other statistical analyses. The health system’s Quality Review Committee approved the study as a QI project.

## RESULTS

### Patient Demographics

There were 266 encounters by 163 patients during the preintervention period and 488 encounters by 238 patients during the intervention period after excluding 22 and 41 transfers in the preintervention and intervention study periods, respectively. Demographics between patients during the preintervention and intervention study periods were similar (Table 1). A higher proportion of patients were admitted to the intensive care unit (ICU) during the preintervention (13.2%) compared to the intervention study periods (8.0%) (*P* = 0.02), and a higher proportion presented to the ED on the weekend during the intervention study period (19.2% versus 28.1%, *P* = 0.01).

**Table 1.** Patient Demographics

	Preimplementation Period (N = 266), n (%)	Implementation Period (N = 488), n (%)	*P*
Age (y)			0.07
<1	22 (8.3)	64 (13.1)	
1–4	72 (27.1)	147 (30.1)	
5–12	100 (37.6)	174 (35.7)	
13–18	72 (27.1)	103 (21.1)	
Sex, male	174 (65.4)	319 (65.4)	0.99
Race/ethnicity*			0.18
White	203 (76.3)	377 (78.5)	
Black	62 (23.3)	98 (20.4)	
Other†	1 (0.4)	5 (1.0)	
Payer			0.23
Private	102 (38.4)	193 (39.6)	
Public	161 (60.5)	287 (58.8)	
Other‡	3 (1.1)	8 (1.6)	
Time of presentation (5 pm–8 am)	154 (57.9)	267 (54.7)	0.40
Day of presentation (weekend)	51 (19.2)	137 (28.1)	0.01
ESI			0.21
1-Resuscitation	1 (0.4)	2 (0.4)	
2-Emergent	110 (41.4)	211 (43.3)	
3-Urgent	153 (57.5)	270 (55.3)	
4-Less urgent	2 (0.8)	4 (0.8)	
5-Non-urgent	0 (0.0)	1 (0.2)	
Operative intervention within 12 h	20 (7.5)	27 (5.5)	0.28
Discharged from the ED	120 (48.5)	209 (42.8)	0.07
ICU admission	35 (13.2)	39 (8.0)	0.02
Encounter defined as possible shunt malfunction§			
Chief complaint	170 (63.9)	305 (62.5)	0.70
ICD10-CM principal diagnosis code	41 (15.4)	99 (20.3)	0.10
Shunt series	232 (87.2)	420 (86.1)	0.66

*Eight patients declined to provide race/ethnicity information and were therefore excluded from these proportions.

†Indian (Asia), multiple races/ethnicities; there were no Hispanic patients in our cohort.

‡Self-pay, Amish, Federal health program supplement.

^§^Sum of proportions is greater than 100%, as an encounter could be identified by more than method.

### Neuroimaging Rates

**Fig. 2.**
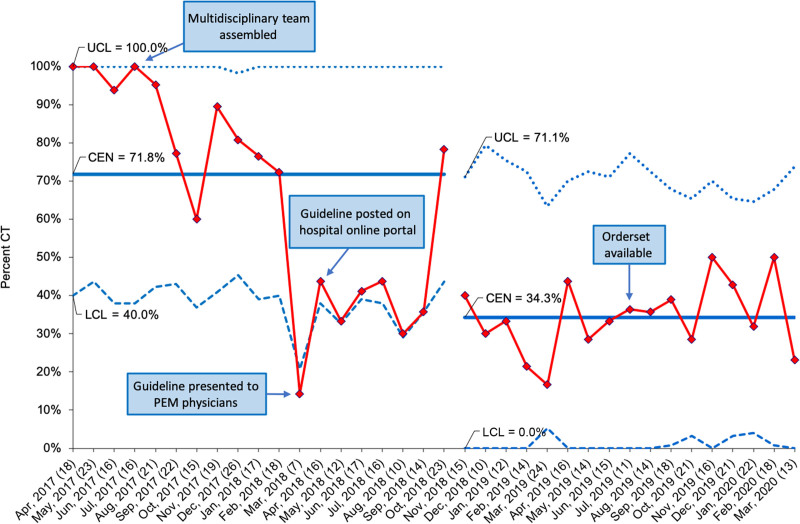
SPC chart (p chart) demonstrating the proportion of encounters with CT among those with neuroimaging for ventricular shunt evaluation, by month. We indicate the number of patients evaluated each month in parenthesis on the *x* axis. CEN, center line (mean); LCL, lower control limit; UCL, upper control limit.

During the preintervention period, and adjusting for repeat encounters across patients, 80.7% of encounters for possible ventricular shunt malfunction included a neuroimaging study, with a similar frequency during the intervention study period (81.5%), (*P* = 0.09) (Table 2). CT imaging frequency decreased significantly from 90.1% to 34.8% during the preintervention and intervention study periods, respectively (difference −55.3% [95% confidence interval (CI): −71.1, −25.8]). Analyzing the data with an SPC chart (Fig. 2) supported the hypothesis that guideline implementation drove this reduction in CT. There was special cause variation toward the study goal detected shortly after implementation.

**Table 2.** Primary Outcome, and Process and Balancing Measures

	Preimplementation Period	Implementation Period	Difference (95% CI)
Neuroimaging, n (%)			
Any	212 (80.7)	394 (81.5)	0.8 (−6.9, 4.6)
CT	183 (90.1)	152 (34.8)	−55.3 (−71.7, −25.8)
rMRI	31 (9.9)	248 (65.2)	55.3 (25.8, 71.1)
Time to neuroimaging (min), mean (SD)	100.2 (56.2)	153.3 (74.7)	53.1 (41.6, 64.6)
ED LOS (min), mean (SD)	230.4 (99.8)	282.6 (104.8)	52.3 (36.8, 67.7)
Time to operative intervention (min), mean (SD)	281.1 (128.5)	384.9 (129.6)	103.8 (16.3, 191.3)
Admitted	235.1 (110.0)	278.3 (102.9)	43.2 (21.5, 65.0)
Discharged	223.3 (88.0)	288.6 (107.6)	65.3 (43.1, 87.4)
Total neuroimaging, n (%)			
0	46 (17.3)	77 (15.8)	−1.5 (−4.8, 5.4)
1	195 (73.3)	359 (73.6)	0.3 (−7.5, 5.4)
2	22 (8.3)	46 (9.4)	1.2 (−1.7, 9.3)
3	3 (1.1)	4 (0.8)	−0.3 (−0.3, 7.0)
4	0 (0)	2 (0.4)	0.4 (−1.0, 0.2)
ED revisit for shunt evaluation within 72 h of index visit, n (%)	3 (2.3)	7 (3.4)	1.0 (−0.5, 23.1)
Follow-up imaging within 7 d, n (%)	8 (6.2)	9 (4.3)	−1.9 (−2.3, 7.8)

There were 2 ED encounters in the preintervention and 5 in the intervention study periods that included both CT and MRI, and in all encounters, the MRI was performed first. Further review of these 7 encounters revealed that in 3 cases, patients did not tolerate the MRI. In 3 cases, neurosurgery requested the CT after the MRI. The reason for the MRI after CT for the last encounter is unknown. When evaluating monthly trends in CT use (Fig. 2), there is a notable rise in CT use back to the near-baseline rate 7 months after guideline implementation, and the study team further investigated these 23 encounters. Specifically, MRI technicians did not note any equipment malfunctions or technical challenges during this time. Upon chart review, there was not a consistent theme for why rMRI-shunt was not performed.

### Balancing Measures

**Fig. 3.**
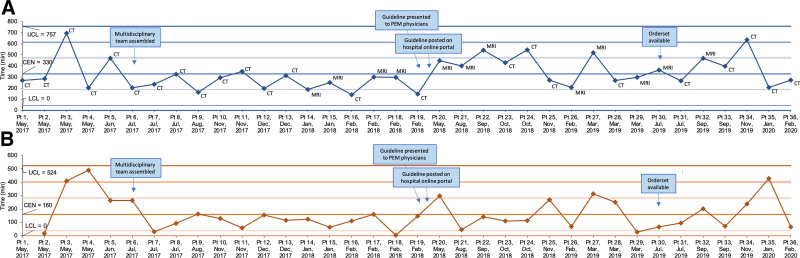
SPC charts demonstrating time to the operating room for those with operative intervention within 12 hours of ED arrival. Individuals chart (A) and moving ranges chart (B). The *x* axis shows each patient and the date of visit. We label each time point on the individual’s chart with the imaging study performed first in the ED. CEN, center line (mean); LCL, lower control limit; UCL, upper control limit.

The mean time to neuroimaging was 100.2 minutes in the preintervention and 153.3 minutes in the intervention study groups (difference 53.1 min [95% CI: 41.6, 64.6] (Table 2). Mean ED LOS was also higher in the intervention group compared to the preintervention study group (difference 52.3 min [95% CI: 36.8, 67.7]) (Table 2). For the 20 and 27 patients who went to the operating room within 12 hours during the preintervention and intervention study periods, respectively, mean times to the operating room were also higher in the intervention group (384.9 min) compared to the preintervention group (281.1 min) (difference 103.8 min [95% CI: 16.3, 191.3]). However, more detailed chronological analyses with individual and moving ranges SPC charts (Fig. 3) did not reveal any special cause variation, suggesting that the intervention did not significantly impact the time to operative intervention.

## DISCUSSION

Through multidisciplinary team development of a standardized, evidence-based guideline, educational outreach to relevant stakeholders, and periodic data sharing with ED providers, we observed a 55% decrease in ED CT use and replacement with rMRI-shunt imaging for children presenting for ventricular shunt evaluation. This decline was sustained for 2 years following implementation. The increase in rMRI-shunt resulted in an increased time to neuroimaging, ED LOS, and time to operative intervention, but no difference in the total number of neuroimaging studies, 72-hour revisits, or 1-week follow-up neuroimaging. It is important to understand these findings in the context of national data. A recent study of 32 pediatric EDs found a 28% rate of MRI for ventricular shunt evaluation in 2018,^21^ well below that during our intervention period, which supports our intervention’s impact.

Children with ventricular shunts are exposed to many radiation-conferring imaging studies over the life of their shunt.^7–9^ The risks of radiation exposure have been highlighted in several epidemiologic studies,^2,3^ and evidence suggests a “dose-dependent” relationship between the number of head CT exposures and malignancy risk.^2,3,22^ Therefore, it is essential for clinicians to consider radiation-sparing alternatives, particularly when evaluating children for shunt malfunctions.

Campaigns, such as Image Gently, strive to promote safe decreases in unnecessary radiation exposure to pediatric patients.^23^ In keeping with this paradigm, and similar to other QI efforts to decrease ED radiation exposure,^24–26^ our findings suggest a rMRI-shunt protocol can be performed instead of CT for the majority of pediatric patients with ventricular shunts. However, the reduction in radiation must be balanced against the clear increase in time required to obtain and perform the rMRI-shunt study and increase other metrics such as ED LOS. Even though the rMRI-shunt shunt protocol requires only 7 minutes of scanning time, obtaining an MRI requires additional time, including a strict screening process, and usually requires transport out of the ED. There was an increase in time to operative intervention. However, readers should interpret these results with caution, and more data would be needed to better understand the impact of the change in imaging strategy on this measure.

Although there was a >50% decline, we can likely decrease CT use in this population even further. One of the next improvement strategies to employ is audit-and-feedback, which has been shown to be of benefit in other QI studies,^24,25^ and may further improve the use of rMRI-shunt in this population. Of note, implementing a standardized order set did not lead to additional improvements in MRI use in this study, possibly because clinicians were already placing rMRI-shunt orders correctly, and/or MRI technicians were proactive in clarifying orders. Our ED uses 3 additional rMRI protocols for various conditions in the ED (stroke, abusive head trauma, and nonspecific neurologic symptoms), which has led to an increase in demand for MRI imaging.^11^ This may have contributed to the apparent MRI ordering threshold we observed in the last year of the study below which further improvements are unlikely to be made. Nonetheless, the goal is to continue to identify residual barriers beyond MRI availability that may be contributing to continued reliance on CT. For example, the treating team often consults neurosurgery residents early in the ED evaluation process. As most of their training is focused on adult patients for whom CT remains the standard of care,^27^ there may be some resistance to MRI. Another consideration is that some patients will not tolerate the rMRI-shunt study, despite its truncated scan duration; it remains longer than that for head CT. It is important to note that the clinical effectiveness guideline recommends that neuroimaging not be delayed in critically ill patients, and a CT would be preferable in these cases. Therefore, the proportion of encounters with CT should never be zero. Although we found that neuroimaging rates were not different between the two study periods, the absolute imaging rate was high. With implementation of novel tools (eg, ShuntCheck^28^), future efforts should focus on safely reducing overall neuroimaging in these patients.

There are limitations to this work. This study was a single-center QI project at a tertiary care children’s hospital ED, and the results may not generalize to other EDs. The higher proportion of patients admitted to the ICU in the preintervention period may suggest a higher acuity patient group and could have contributed to the higher CT rate during this period. However, there was no difference in emergency severity index between the 2 groups, and, furthermore, this finding alone would be unlikely to explain the magnitude of change in CT observed. Our study was not designed to evaluate downstream effects of our intervention, including impact on overall patient flow and LOS, and these data will be necessary for future work. Data were limited to imaging studies and visits within the study hospital, and assessments of revisits and follow-up neuroimaging could have been affected if a patient sought care at another ED. However, this limitation’s impact is likely to be small, as the study ED is the only pediatric ED in the region and prior work suggests most ED revisits will be to the index ED.^29^ Finally, this study did not measure parental/guardian preferences regarding CT versus MRI risks and benefits for their child which deserves further study.

## CONCLUSIONS

Implementation of a multidisciplinary, evidence-based guideline emphasizing the use of rMRI-shunt as the ED neuroimaging study of choice for most children with suspected ventricular shunt malfunction resulted in a 50% reduction in CT. Reducing radiation exposure by increasing rMRI-shunt use should be balanced against the increased time needed to perform rMRI-shunt imaging. Future work should focus on improvements to further reduce the reliance on CT and continued improvements in rMRI-shunt technology to reduce scanning time and increase resource availability.

## DISCLOSURE

The authors have no financial interest to declare in relation to the content of this article.

## Supplementary Material



## References

[1] Sivaganesan A Krishnamurthy R Sahni D. Neuroimaging of ventriculoperitoneal shunt complications in children. Pediatr Radiol. 2012;42:1029–1046. 22740019 10.1007/s00247-012-2410-6

[2] Pearce MS Salotti JA Little MP. Radiation exposure from CT scans in childhood and subsequent risk of leukaemia and brain tumours: a retrospective cohort study. Lancet. 2012;380:499–505. 22681860 10.1016/S0140-6736(12)60815-0 PMC3418594

[3] Mathews JD Forsythe AV Brady Z. Cancer risk in 680,000 people exposed to computed tomography scans in childhood or adolescence: data linkage study of 11 million Australians. BMJ. 2013;346:f2360. 23694687 10.1136/bmj.f2360 PMC3660619

[4] Stone JJ Walker CT Jacobson M. Revision rate of pediatric ventriculoperitoneal shunts after 15 years. J Neurosurg Pediatr. 2013;11:15–19. 23101557 10.3171/2012.9.PEDS1298

[5] Browd SR Ragel BT Gottfried ON. Failure of cerebrospinal fluid shunts: part I: obstruction and mechanical failure. Pediatr Neurol. 2006;34:83–92. 16458818 10.1016/j.pediatrneurol.2005.05.020

[6] Garton HJ Kestle JR Drake JM. Predicting shunt failure on the basis of clinical symptoms and signs in children. J Neurosurg. 2001;94:202–210. 11213955 10.3171/jns.2001.94.2.0202

[7] Antonucci MC Zuckerbraun NS Tyler-Kabara EC. The burden of ionizing radiation studies in children with ventricular shunts. J Pediatr. 2017;182:210–216.e1. 27989409 10.1016/j.jpeds.2016.11.051

[8] Florin TA Aronson PL Hall M. Emergency department use of computed tomography for children with ventricular shunts. J Pediatr. 2015;167:1382–1388.e2. 26474707 10.1016/j.jpeds.2015.09.024

[9] Cohen JS Jamal N Dawes C. Cranial computed tomography utilization for suspected ventriculoperitoneal shunt malfunction in a pediatric emergency department. J Emerg Med. 2014;46:449–455. 24472355 10.1016/j.jemermed.2013.08.137

[10] Sodickson A Baeyens PF Andriole KP. Recurrent CT, cumulative radiation exposure, and associated radiation-induced cancer risks from CT of adults. Radiology. 2009;251:175–184. 19332852 10.1148/radiol.2511081296

[11] Ramgopal S Karim SA Subramanian S. Rapid brain MRI protocols reduce head computerized tomography use in the pediatric emergency department. BMC Pediatr. 2020;20:14. 31931764 10.1186/s12887-020-1919-3 PMC6956479

[12] Ashley WW Jr McKinstry RC Leonard JR. Use of rapid-sequence magnetic resonance imaging for evaluation of hydrocephalus in children. J Neurosurg. 2005;103(2 suppl):124–130. 10.3171/ped.2005.103.2.0124 16370277

[13] Flom L Fromkin J Panigrahy A. Development of a screening MRI for infants at risk for abusive head trauma. Pediatr Radiol. 2016;46:519–526. 26589303 10.1007/s00247-015-3500-z PMC4814308

[14] Missios S Quebada PB Forero JA. Quick-brain magnetic resonance imaging for nonhydrocephalus indications. J Neurosurg Pediatr. 2008;2:438–444. 19035694 10.3171/PED.2008.2.12.438

[15] Yue EL Meckler GD Fleischman RJ. Test characteristics of quick brain MRI for shunt evaluation in children: an alternative modality to avoid radiation. J Neurosurg Pediatr. 2015;15:420–426. 25634816 10.3171/2014.9.PEDS14207

[16] Boyle TP Paldino MJ Kimia AA. Comparison of rapid cranial MRI to CT for ventricular shunt malfunction. Pediatrics. 2014;134:e47–e54. 24918222 10.1542/peds.2013-3739

[17] ICD10Data.Com. Available at https://www.icd10data.com/ICD10CM/Codes/S00-T88/T80-T88/T85-. Published 2018. Accessed May 14, 2020.

[18] Wang L Zhou H Zhu JF. Application of emergency severity index in pediatric emergency department. World J Emerg Med. 2011;2:279–282. 25215023 10.5847/wjem.j.1920-8642.2011.04.006 PMC4129726

[19] Austin PC Merlo J. Intermediate and advanced topics in multilevel logistic regression analysis. Stat Med. 2017;36:3257–3277. 28543517 10.1002/sim.7336 PMC5575471

[20] Provost LP Murray S. The Health Care Data Guide: Learning From Data for Improvement. Jossey-Bass; 2011.

[21] Marin JR Rodean J Hall M. Trends in use of advanced imaging in pediatric emergency departments, 2009-2018. JAMA Pediatric. 2020;174:e202209–2. 10.1001/jamapediatrics.2020.2209 PMC7400208 32761186

[22] Brenner D Elliston C Hall E. Estimated risks of radiation-induced fatal cancer from pediatric CT. AJR Am J Roentgenol. 2001;176:289–296. 11159059 10.2214/ajr.176.2.1760289

[23] Strauss KJ Goske MJ Kaste SC. Image gently: ten steps you can take to optimize image quality and lower CT dose for pediatric patients. AJR Am J Roentgenol. 2010;194:868–873. 20308484 10.2214/AJR.09.4091

[24] Jennings RM Burtner JJ Pellicer JF. Reducing head CT use for children with head injuries in a community emergency department. Pediatrics. 2017;139:e20161349. 28255067 10.1542/peds.2016-1349

[25] Nigrovic LE Stack AM Mannix RC. Quality improvement effort to reduce cranial CTs for children with minor blunt head trauma. Pediatrics. 2015;136:e227–e233. 26101363 10.1542/peds.2014-3588 PMC5660895

[26] Marchese RF Schwartz ES Heuer GG. Reduced radiation in children presenting to the ED with suspected ventricular shunt complication. Pediatrics. 2017;139:e20162431. 28557725 10.1542/peds.2016-2431

[27] Lehnert BE Rahbar H Relyea-Chew A. Detection of ventricular shunt malfunction in the ED: relative utility of radiography, CT, and nuclear imaging. Emerg Radiol. 2011;18:299–305. 21523469 10.1007/s10140-011-0955-6

[28] Madsen JR Boyle TP Neuman MI. Diagnostic accuracy of non-invasive thermal evaluation of ventriculoperitoneal shunt flow in shunt malfunction: a prospective, multi-site, operator-blinded study. Neurosurgery. 2020;31:435. 10.1093/neuros/nyaa128 PMC7566379 32459841

[29] Lyons TW Olson KL Palmer NP. Patients visiting multiple emergency departments: patterns, costs, and risk factors. Acad Emerg Med. 2017;24:1349–1357. 28861915 10.1111/acem.13304 PMC5681430

