# Supplementary material for: Replacing Computed Tomography with “Rapid” Magnetic Resonance Imaging for Ventricular Shunt Imaging
Source: Pediatr Qual Saf. 2021 Jul 28;6(4):e441. doi: 10.1097/pq9.0000000000000441 (PMC8322500; doi:10.1097/pq9.0000000000000441)
Supplement: Supplementary file 1 [file pqs-6-e441-s001.pdf]

Table: Rapid MRI Ventricular Shunt Imaging Protocol

**MRI Sequence**	**Duration**
SAGITTAL T2 SSFSE	1 min
AXIAL T2 SSFSE	1 min
AXIAL GRE	1 min 45 sec

MRI= magnetic resonance imaging

SSFSE= single shot fast spin echo

GRE=gradient echo
