# Supplementary material for: Replacing Computed Tomography with “Rapid” Magnetic Resonance Imaging for Ventricular Shunt Imaging
Source: Pediatr Qual Saf. 2021 Jul 28;6(4):e441. doi: 10.1097/pq9.0000000000000441 (PMC8322500; doi:10.1097/pq9.0000000000000441)
Supplement: Supplementary file 2 [file pqs-6-e441-s002.pdf]

ED Imaging, Concern for Ventricular Shunt Malfunction

UPMC CHILDREN'S HOSPITAL OF PITTSBURGH

CLINICAL EFFECTIVENESS GUIDELINE

Emergency Department Imaging for Patients with Concern for Ventricular Shunt Malfunction

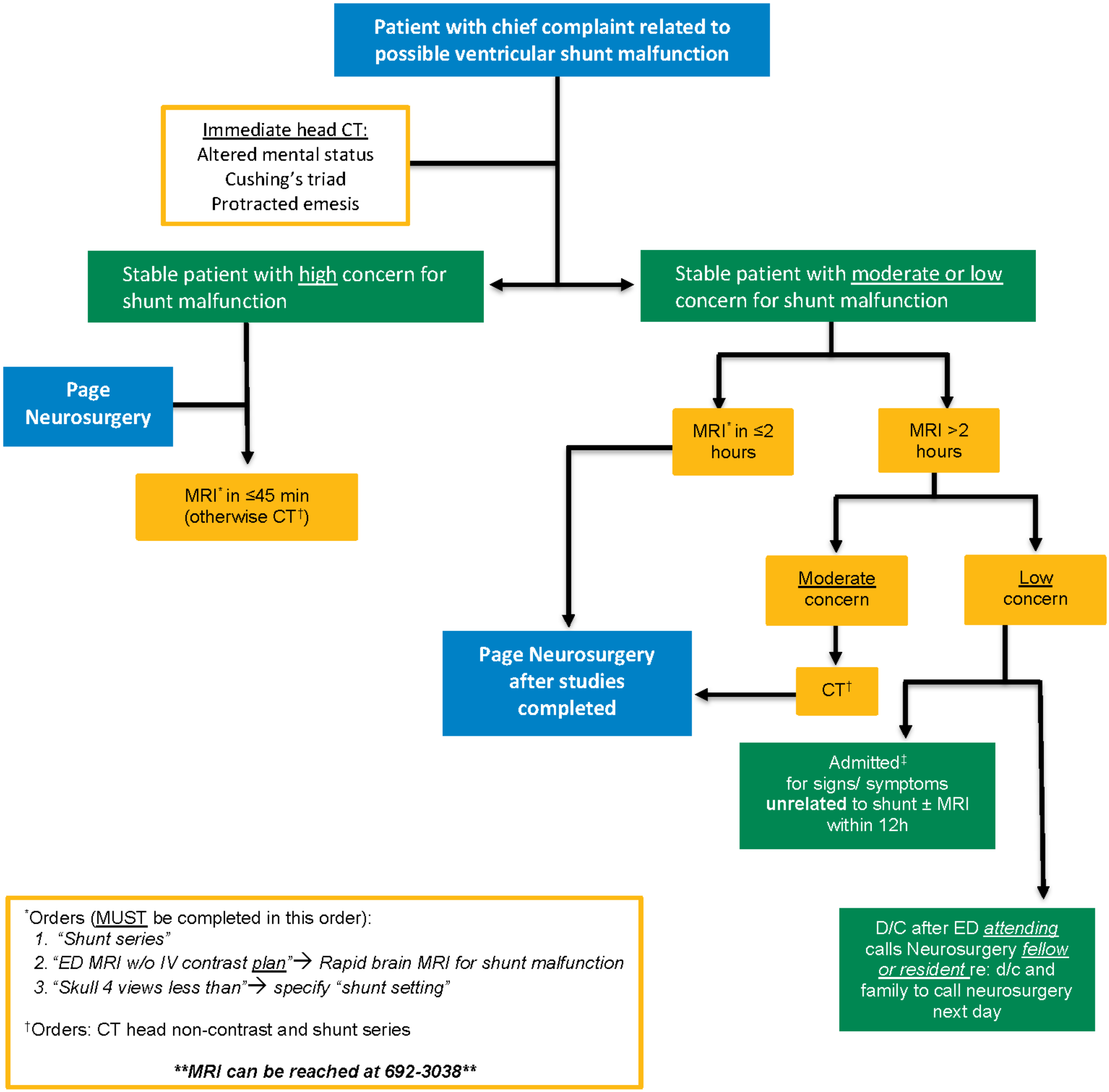

*Orders (MUST be completed in this order):

1. *"Shunt series"*
2. *"ED MRI w/o IV contrast plan"→ Rapid brain MRI for shunt malfunction*
3. *"Skull 4 views less than"→ specify "shunt setting"*

†Orders: CT head non-contrast and shunt series

*****MRI can be reached at 692-3038*****
